# The correlation between trimethylamine N-oxide, lipoprotein ratios, and conventional lipid parameters in patients with unstable angina pectoris

**DOI:** 10.1042/BSR20192657

**Published:** 2020-01-14

**Authors:** Zeng-Xiang Dong, Jia Zhang, Ying-Chun Luo, Ming-Ming Zhao, Jia-Geng Cai, Si Cheng, Le-Min Zheng, Xin Hai

**Affiliations:** 1 Department of Pharmacy, the First Affiliated Hospital of Harbin Medical University, Harbin, China; 2 Department of Cardiology, the First Affiliated Hospital of Harbin Medical University, Harbin, China; 3 The Institute of Cardiovascular Sciences and Institute of Systems Biomedicine, School of Basic Medical Sciences, Peking University Health Science Center, Key Laboratory of Molecular Cardiovascular Science of Ministry of Education, Key Laboratory of Cardiovascular Molecular Biology and Regulatory Peptides of Ministry of Health, Beijing Key Laboratory of Cardiovascular Receptors Research, Beijing 100191, China; 4 Tianjin Key Laboratory of Ionic-Molecular Function of Cardiovascular Disease, Department of Cardiology, Tianjin Institute of Cardiology, the Second Hospital of Tianjin Medical University, Tianjin 300211, China; 5 China National Clinical Research Center for Neurological Diseases, Tiantan Hospital, Advanced Innovation Center for Human Brain Protection, The Capital Medical University, Beijing 100050, China

**Keywords:** Unstable angina pectoris, Trimethylamine N-oxide, Lipoprotein ratios, Risk factors

## Abstract

Purpose: Trimethylamine N-oxide (TMAO) is recently the main risk factor for coronary heart disease (CHD). Plasma lipid levels are conventionally used to predict coronary risk, but the correlation between TMAO and plasma lipid levels in unstable angina pectoris (UAP) was unclear. Our objective was to compare the plasma level of TMAO to lipoprotein ratios and conventional lipid parameters in UAP patients. Methods: A total of 114 control participants and 184 UAP patients were enrolled. Demographic characteristics were collected. Plasma levels of TMAO and lipid in all patients were measured and analyzed. The receiver operating characteristic analysis (ROC), univariate, and multivariate logistic regression analyses were carried out to examine the relationship between TMAO, lipoprotein ratios, conventional lipid parameters, and UAP. Results: The plasma levels of TMAO were remarkably increased in UAP patients (3.28 ± 1.97 µM) compared with control participants (1.52 ± 0.59 µM, *P* < 0.01). TMAO was significantly correlated with lipid levels in UAP patients. The ROC, univariate and multivariate logistic regression analysis both showed that the TMAO significantly increased the risk for occurrence of UAP. Conclusions**:** Our data indicate that the TMAO is superior to lipoprotein ratios and conventional lipid parameters in predicting occurrence of UAP.

## Introduction

Coronary heart disease (CHD) has become a global health problem and places a huge burden on the healthcare system in developed and developing countries [1–3]. CHD comprises acute myocardial infarction (AMI), stable angina pectoris (SAP), unstable angina pectoris (UAP), and sudden cardiac death [4]. UAP is associated with higher risk of morbidity and mortality than SAP [5]. It is of great clinical importance to assess risk of UAP by reliable method for managing and preventing this disease. At present, many studies have demonstrated that the plasma level of total cholesterol (TC), low-density lipoprotein cholesterol (LDL-C), high-density lipoprotein cholesterol (HDL-C), triglyceride (TG), apolipoprotein A1 (ApoA1) and apolipoprotein B 100 (ApoB100) are commonly used as CHD predictors in clinical practice. Nevertheless, there are many arguments about coronary risk assessment based exclusively on conventional lipid parameters are not optimal [6–8]. Efforts have been made in seeking emergent or new cardiovascular risk markers to improve CHD prediction, especially for UAP. At the present time, lipoprotein ratios including ApoB100/ApoA1, TC/HDL-C, TG/HDL-C, and LDL-C/HDL-C are used as CHD predictors in clinical practice [9].

Trimethylamine N-oxide (TMAO) is a plasma metabolite from nutrient precursors (choline, phosphatidylcholine, and L-carnitine derived mainly from red meat, egg yolks, dairy products, and seafood), which is produced by gut microbiota [10–12]. Many studies have revealed a relation between plasma levels of TMAO and cardiovascular risks. These studies have indicated that plasma levels of TMAO were associated with risk of major adverse cardiac events independent of traditional risk factors [13–17]. Our previous study found that plasma levels of TMAO were an independent predictor of CHD in patients [18]. However, the predictive capability between TMAO levels and lipoprotein ratios for UAP in patients has not yet been compared. Thus, we aimed to assess the relationship between TMAO levels, lipoprotein ratios, and UAP risk in patients.

## Methods

### Participants

Between May 2016 and August 2017, 298 participants were enrolled in the First Affiliated Hospital of the Harbin Medical University (Harbin, China). The study consisted of two groups: control participants (*n* = 114) and UAP patients (*n* = 184). All participants underwent a complete physical examination, and the medical history of all participants was recorded. Control participants were patients with negative medical history of UAP. The diagnosis of UAP was confirmed by cardiologist, presented as chest pain at rest or aggravated effort type angina within 1 month with the changes of definite ischemic electrocardiogram or recurrent angina pectoris [5]. Exclusion criteria included patients with any known chronic disease, cardiac valve diseases, congenital heart diseases, diagnosed pulmonary disease, aortic aneurysm, connective tissue diseases, and cancer. The clinical characteristics of the study population are summarized in Table 1. The study protocols and the procedures for handling human samples were approved by the Institutional Research Board of the Harbin Medical University. The research has been carried out in accordance with the World Medical Association Declaration of Helsinki, and that all subjects provided written informed consent.

**Table 1:** Baseline characteristics of the study population

Characteristic	Control (*n* = 114)	UAP (*n* = 184)	*P* value
Age (years)	52.2 ± 14.9	59.1 ± 9.3	<0.01
Male (%)	38 (33.3%)	87(47.3%)	0.018
HDL-C (mmol/l)	1.30 ± 0.31	1.29 ± 0.32	0.793
LDL-C (mmol/l)	2.82 ± 0.68	2.93 ± 0.76	0.188
Total cholesterol (mmol/l)	4.73 ± 1.02	4.89 ± 1.15	0.238
Triglyceride (mmol/l)	1.70 ± 1.06	1.87 ± 1.07	0.167
ApoA1 (g/l)	1.28 ± 0.24	1.27 ± 0.25	0.560
ApoB100 (g/l)	0.98 ± 0.30	1.04 ± 0.37	0.117
ApoB100 / ApoA1	0.78 ± 0.26	0.87 ± 0.44	0.057
TC/HDL-C	3.78 ± 0.93	3.93 ± 1.01	0.201
TG/HDL-C	1.45 ± 1.25	1.58 ± 0.98	0.337
LDL-C/HDL-C	2.26 ± 0.65	2.36 ± 0.69	0.208
hs-TnI (ng/ml)	0.011 ± 0.003	0.324 ± 0.138	0.028
CKMB (ng/ml)	1.00 ± 0.08	2.51 ± 0.83	0.231

Data are presented as mean ± standard deviation or proportions; HDL-C, high-density lipoprotein cholesterol; LDL-C, low-density lipoprotein cholesterol; ApoA1, apolipoprotein A1; ApoB100, apolipoprotein B100; hs-TnI, high-sensitive troponin I; CKMB, creatine kinase MB.

### Collection and handling of human blood samples

The biochemical variables, including the plasma level of total cholesterol (TC), low-density lipoprotein cholesterol (LDL-C), high-density lipoprotein cholesterol (HDL-C), triglyceride (TG), apolipoprotein A1 (ApoA1), apolipoprotein B100 (ApoB100), high-sensitive troponin I (hs-TNI), and creatine kinase-MB (CK-MB) were tested in the clinical laboratory of the First Affiliated Hospital of the Harbin Medical University. For determination of TMAO, whole blood samples (1 ml per patient) were obtained from the study subjects via a direct venous puncture into the tubes containing sodium citrate. The whole blood samples were centrifuged at 2000 × *g*/min at 4°C for 10 min to obtain plasma samples.

### Determination of TMAO

We measured TMAO by LC-MS/MS using TMAO-d9 (TRC, Canada) as internal standards according to our previous method [18]. Berifly, 5 μl of each sample supernatant was determined by an Agilent 1100 high performance liquid chromatography (HPLC) system (Agilent Technologies, U.S.A.), and analytes were separated on a phenomenex Luna Silica column (100 mm × 2 mm, 3 μm particle size) at room temperature. API 4000 triple quadrupole mass spectrometer (AB Sciex, U.S.A.) was uesd to determine the level of TMAO. Ion transitions used for quantitation were *m/z* 76 → 58 for TMAO and *m*/*z* 85 → 66 for the internal standards.

### Statistical analysis

Categorical variables were presented as frequency and continuous variables were expressed as mean ± SD (standard deviation). Differences between groups in categorical variables were analyzed using the chi-squared test and in continuous variables using the unpaired two-tailed *t* test. The receiver operating characteristic (ROC) curves were applied and the area under the curve (AUC) were determined to evaluate UAP risk. Univariate and multivariate logistic regression analysis were used to assess the association between the TMAO, lipoprotein ratios, and UAP risks. The correlation analysis for the association of TMAO with lipid parameters was measured by Pearson’s for continuous data or Spearman’s for categorical data. All analyses were performed using SPSS v20.0 software. Statistical significance was accepted at *P* < 0.05 for all analyses.

## Results

### Clinical characteristics of the study population

The study consisted of two subject groups: 114 control participants and 184 UAP patients. Table 1 shows the clinical and demographic characteristics of the patients enrolled in the present study. In the all subject groups, there was significant difference in the age, sex distribution and hs-TNI between UAP patients compared with controls (*P* < 0.05).

### The levels of TMAO in control participants and UAP patients

**Figure 1:**
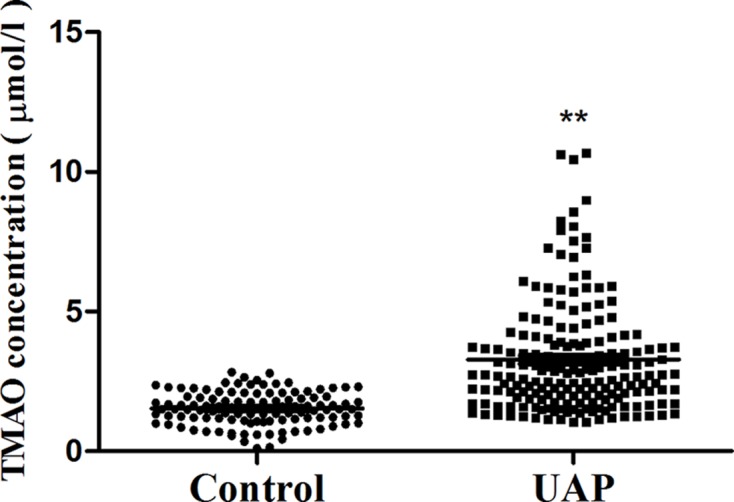
The plasma levels of TMAO The levels of TMAO were confirmed in control and UAP group. **P<0.01, control vs. UAP group.

LC-MS/MS analysis was used to determine the concentration of TMAO in plasma. As Figure 1 shows that there was significant difference in plasma levels of TMAO between control and UAP group. The levels of TMAO were significantly higher in UAP patients than that in control group (3.28 ± 1.97 µM vs. 1.52 ± 0.59 µM; *P* < 0.05).

### ROC analysis for evaluation of the correlation between TMAO, lipoprotein ratios, and UAP risk

**Figure 2:**
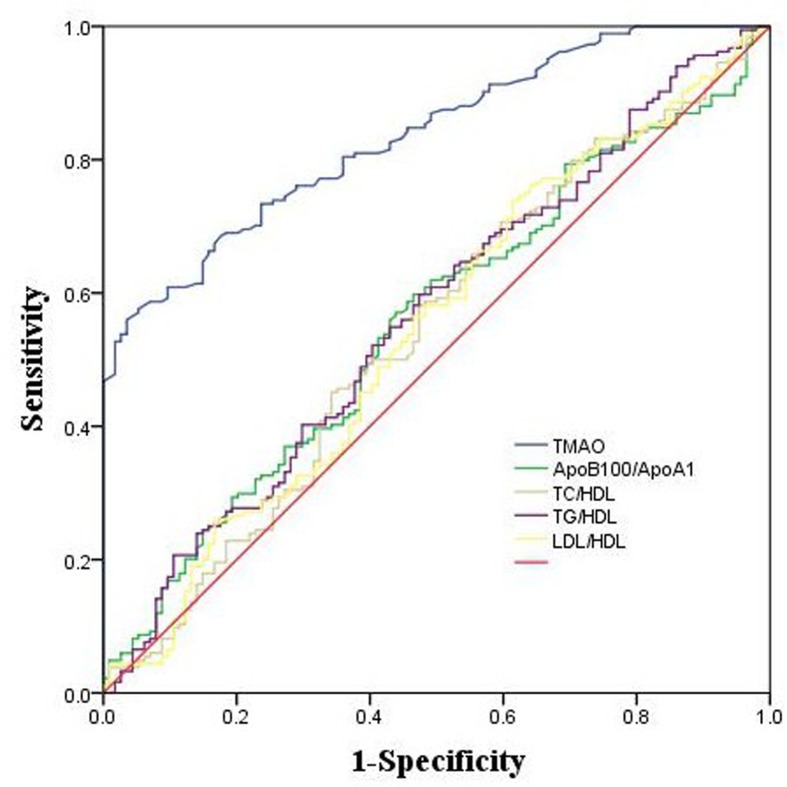
ROC curve of the predictive value of TMAO and lipoprotein ratios for the presence of UAP The area under ROC curve was determined to evaluate the predictive power of circulating TMAO and lipoprotein ratios for UAP.

To compare the predictive power of TMAO and lipoprotein ratios for UAP risk, ROC analysis was performed and AUC was determined. Our results showed that the area under ROC curve of TMAO was 0.835 (95% CI = 0.792–0.878) for UAP (Figure 2). The area under ROC curve of ApoB100/ApoA1, TC/HDL-C, TG/HDL-C, and LDL-C/HDL-C were 0.556 (95% CI = 0.489–0.622), 0.544 (95% CI = 0.476–0.612), 0.566 (95% CI = 0.499–0.633), and 0.546 (95% CI = 0.478–0.614), respectively.

### Univariate and multivariate logistic regression analysis for the correlation between TMAO, lipoprotein ratios, and UAP risk

The univariate logistic regression analysis showed that the odds ratios (OR) were 5.534 (95% CI: 3.497–8.758) for TMAO (*P* < 0.01), between control and UAP group, significantly increased the risk for UAP (Table 2). The univariate logistic regression analyses showed that the ratios of ApoB100/ApoA1, TC/HDL-C, TG/HDL-C, and LDL-C/HDL-C with the OR and 95% CI of 2.111 (0.956–4.657), 1.174 (0.918–1.502), 1.118 (0.889–1.407), and 1.255 (0.880–1.790), respectively (Table 2). No significant association was found between the apolipoprotein parameters and UAP risk in our study population. Lipoprotein ratios were also not associated with UAP risk by univariate logistic regression analysis. Table 3 shows the ORs and 95% CI for UAP as computed by multivariate logistic regression analyses. TMAO were independently associated with UAP after adjustment for age and sex in model 1. These results were not appreciably attenuated with the inclusion of HDL-C, LDL-C, TC, TG, hs-TNI, and CKMB in subsequent models 2 and 3, indicating that these variables did not act as mediators of the relationship between the TMAO and UAP. The association of TMAO with UAP was stronger than lipoprotein ratios.

**Table 2:** Univariate logistic regression analysis for TMAO and lipoprotein ratios with UAP risk

Characteristic	OR	95%CI	*P* value
Age	1.050	1.028–1.073	<0.01
Male	1.688	1.041–2.736	0.034
HDL-C	0.906	0.437–1.879	0.792
LDL-C	1.245	0.898–1.726	0.189
Total cholesterol	1.139	0.918–1.413	0.238
Triglyceride	1.181	0.931–1.499	0.171
ApoA1	0.748	0.283–1.978	0.559
ApoB100	1.805	0.856–3.805	0.121
ApoB100 / ApoA1	2.111	0.956–4.657	0.064
TC/HDL-C	1.174	0.918–1.502	0.202
TG/HDL-C	1.118	0.889–1.407	0.339
LDL-C/HDL-C	1.255	0.880–1.790	0.209
hs-TnI	1.996	0.169–2.351	0.021
CKMB	1.304	0.929–1.831	0.125
TMAO	5.534	3.497–8.758	<0.01

Note: OR, odds ratio; CI, confidence interval; HDL-C, high-density lipoprotein cholesterol; LDL-C, low-density lipoprotein cholesterol; ApoA1, apolipoprotein A1; ApoB, apolipoprotein B100; hs-TnI, high-sensitive troponin I; CKMB, creatine kinase MB.

**Table 3:** Multivariate logistic regression analysis for TMAO and lipoprotein ratios with UAP risk

Characteristic	Model 1		Model 2		Model 3	
	Adjusted OR (95% CI)	*P*	Adjusted OR (95% CI)	*P*	Adjusted OR (95% CI)	*P*
TMAO	5.481(3.368–8.920)	<0.01	5.721(3.470–9.434)	<0.01	5.605(3.190–9.849)	<0.01
ApoB100/ApoA1	1.476(0.655–3.327)	0.348	1.680(0.535–5.269)	0.374	1.504(0.378–5.991)	0.563
TC/HDL-C	1.052(0.806-1.374)	0.708	0.684(0.272–1.717)	0.419	0.390(0.122–1.244)	0.112
TG/HDL-C	1.054(0.829–1.339)	0.669	0.557(0.244–1.274)	0.166	0.440 (0.171–1.135)	0.089
LDL-C/HDL-C	1.036(0.708–1.517)	0.855	0.566(0.151–2.119)	0.398	0.237(0.045–1.259)	0.091

The associations between the variables and UAP were analyzed by multivariate logistic regression; Model 1 - adjusted for age and sex; Model 2 - adjusted for variables in Model 1 plus HDL-C, LDL-C, TC, and TG; Model 3 - adjusted for variables in Model 2 plus hs-TNI and CKMB; OR, odds ratio; CI, confidence interval.

### Correlation of TMAO with other clinical parameters

To identify possible correlations between TMAO level and other clinical parameters for UAP, Pearson’s or Spearman’s correlation analysis was performed. The results showed that TMAO was significantly correlated with age, LDL-C, TC, ApoB100, LDL-C/HDL-C (*P* < 0.05; Table 4). The result indicated that TMAO was significantly correlated with lipid parameters in UAP patients.

**Table 4:** Correlation analysis for the association of TMAO with risk factors for UAP

	TMAO	
	Coefficient	*P* value
Age	0.315	<0.01
Male	0.063	0.395
HDL-C	0.009	0.903
LDL-C	0.219	<0.01
Total cholesterol	0.195	<0.01
Triglyceride	0.053	0.479
ApoA1	0.035	0.633
ApoB100	0.205	<0.01
ApoB100 / ApoA1	0.108	0.144
TC/HDL-C	0.137	0.065
TG/HDL-C	0.002	0.979
LDL-C/HDL-C	0.159	0.032
hs-TnI	0.035	0.646
CKMB	0.042	0.610

## Discussion

The purpose of the present study was to compare the association between the level of TMAO, lipoprotein ratios, and UAP risk. To our knowledge, there are few studies that focused on the relation among level of TMAO, lipoprotein ratios, and UAP risk in patients. In the present study, we found that TMAO was a significant risk factor for UAP. The further information had been demonstrated that the TMAO was better than lipoprotein ratios and conventional lipid parameters in predicting occurrence of UAP, which was based on statistical analysis of clinical variables.

Previous study had indicated that lipoprotein ratios could comprehensively reflect the balance between the atherogenic and antiatherogenic potentials in patients. There is growing evidence that the HDL-C related ratios, including TC/HDL-C, LDL-C/HDL-C, non-HDL-C/HDL-C, VLDL-C/HDL-C and TG/HDL-C, are superior to conventional lipid parameters as predictors for CHD [19–21]. These ratios can provide more information on risk that is difficult to be quantified by routine analyses. They can indicate the metabolic and clinical interactions between lipid fractions. Our previous study found that TMAO could be a risk factor for CHD in Chinese patients [18]. In our previous study, we also demonstrated that plasma levels of TMAO were significantly elevated in CHD patients. TMAO was able to discriminate CHD patients from control participants. The recent study found that the lipoprotein ratios were better than conventional lipid parameters in predicting CHD in Chinese people [9]. In the recent study, the data indicated that the lipoprotein ratios were superior to conventional lipid parameters as predictors for CHD. Of the ratios, ApoB100/ApoA1 was the best to predict CHD risk. Our study was consistant with the above study about ApoB100/ApoA1 was better than other lipoprotein ratios. In our study, the univariate logistic regression analyses showed that the ratios of ApoB100/ApoA1, TC/HDL-C, TG/HDL-C, and LDL-C/HDL-C with the OR and 95% CI of 2.111 (0.956–4.657), 1.174 (0.918–1.502), 1.118 (0.889–1.407), and 1.255 (0.880–1.790), respectively. Furthermore, according to ROC and logistic regression analyses, our data indicated that TMAO was superior to lipoprotein ratios and conventional lipid parameters in predicting UAP with ROC curve of TMAO (AUC: 0.835, 95% CI = 0.792–0.878) and OR (5.534, 95% CI: 3.497–8.758; *P* < 0.01).

Our study has several limitations. First, there was a single center observational and retrospective study. Second, the number of patients who were included in the study was a limitation. Third, prospective data regarding the TMAO and mortality of UAP patients are awaited. Fourth, body mass index was not calculated. We will improve it in subsequent experiments.

In conclusion, to the best of our knowledge, we are able to show for the first time that TMAO is an important risk factor for predicting UAP in patients beyond lipoprotein ratios and conventional lipid parameters. This result indicates that measurement of TMAO levels can be a valuable clue for assessing UAP risk. A better understanding of the relationship between TMAO and UAP will be of great benefit for reducing the incidence of UAP.

## Abbreviations

AUC: area under the curve
CHD: coronary heart disease
ROC: receiver operating characteristic analysis
TMAO: trimethylamine N-oxide
UAP: unstable angina pectoris
